# Supplementary material for: Contemporary Antiretroviral Therapy Dysregulates Iron Transport and Augments Mitochondrial Dysfunction in HIV-Infected Human Microglia and Neural-Lineage Cells
Source: Int J Mol Sci. 2023 Jul 31;24(15):12242. doi: 10.3390/ijms241512242 (PMC10419149; doi:10.3390/ijms241512242)
Supplement: Supplementary file 1 [file ijms-24-12242-s001.zip › ijms-2442460-supplementary.pdf]

Supplemental Materials

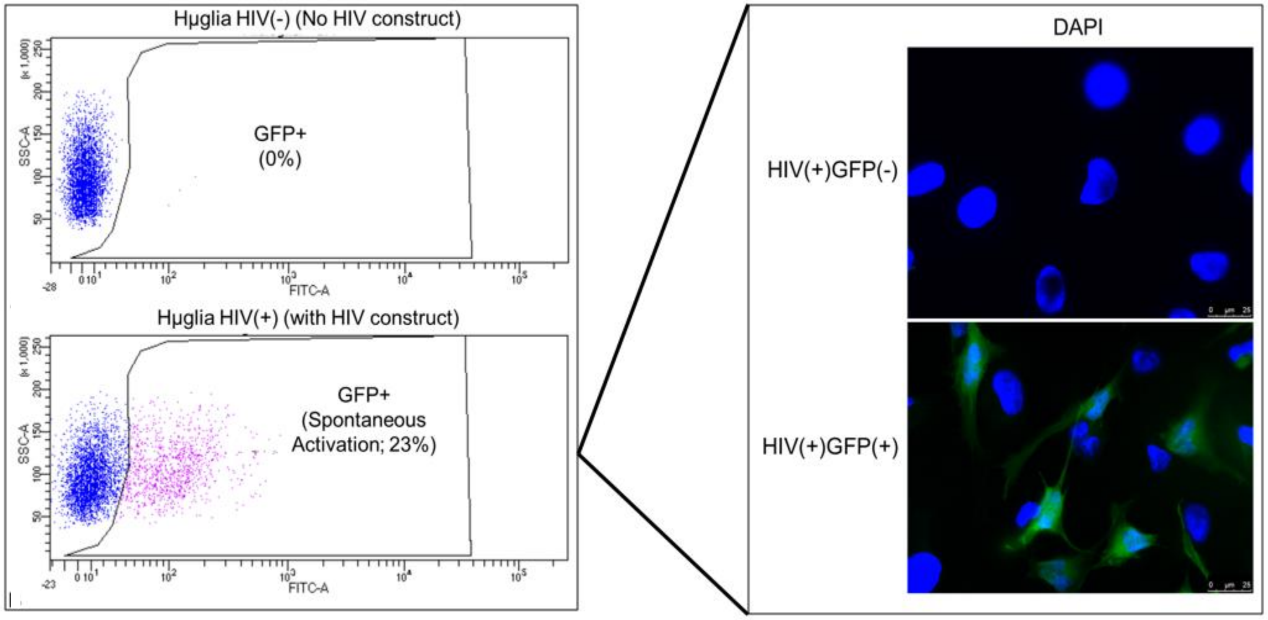

Figure S1. FACS analysis of HIV+ hµglia cell populations. Scatter plot ***(top left panel)*** shows no GFP expression in uninfected hµglia; the ***bottom left panel*** shows HIV+ (infected) C20 hµglia sorted into GFP-negative (blue dots) and GFP+ (pink dots). X-axis shows mean fluorescence intensity of fluorescein isothiocyanate (FITC)-A signal within HIV+ hµglia; Y-axis shows signal gated according to side-scatter-area (SS-A). GFP+ and GFP-negative cell populations were sorted and separated for use in later experiments Green fluorescence in these populations is shown in the ***top right panel*** (HIV+ hµglia containing latent HIV provirus have blue nuclei stained with DAPI and are GFP-negative) and ***bottom right*** (activated HIV+/GFP+ hµglia with ongoing HIV transcription show green fluorescence).

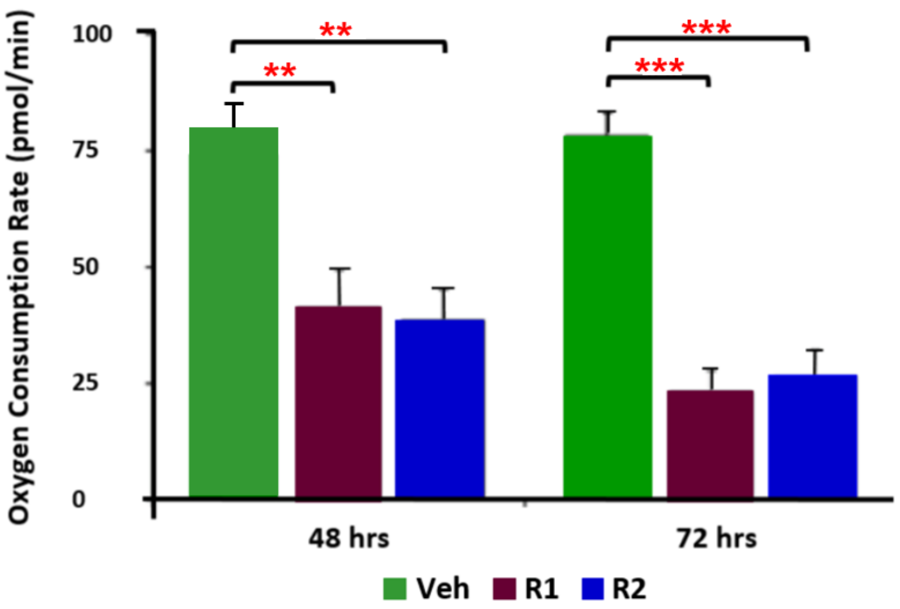

Figure S2. Effects of antiretroviral Regimen1 (*R1*) and 2 (*R2*) on the basal oxygen consumption rate (OCR) in SH-SY5Y cells at 48 and 72 hours. Both R1 and R2 significantly reduce the basal OCR at 72 hours compared to vehicle-treated (*Veh*) controls. Results summarizing at least 3 separate experiments are shown. ***p*<0.01; ****p*<0.001 (Mann-Whitney U test)

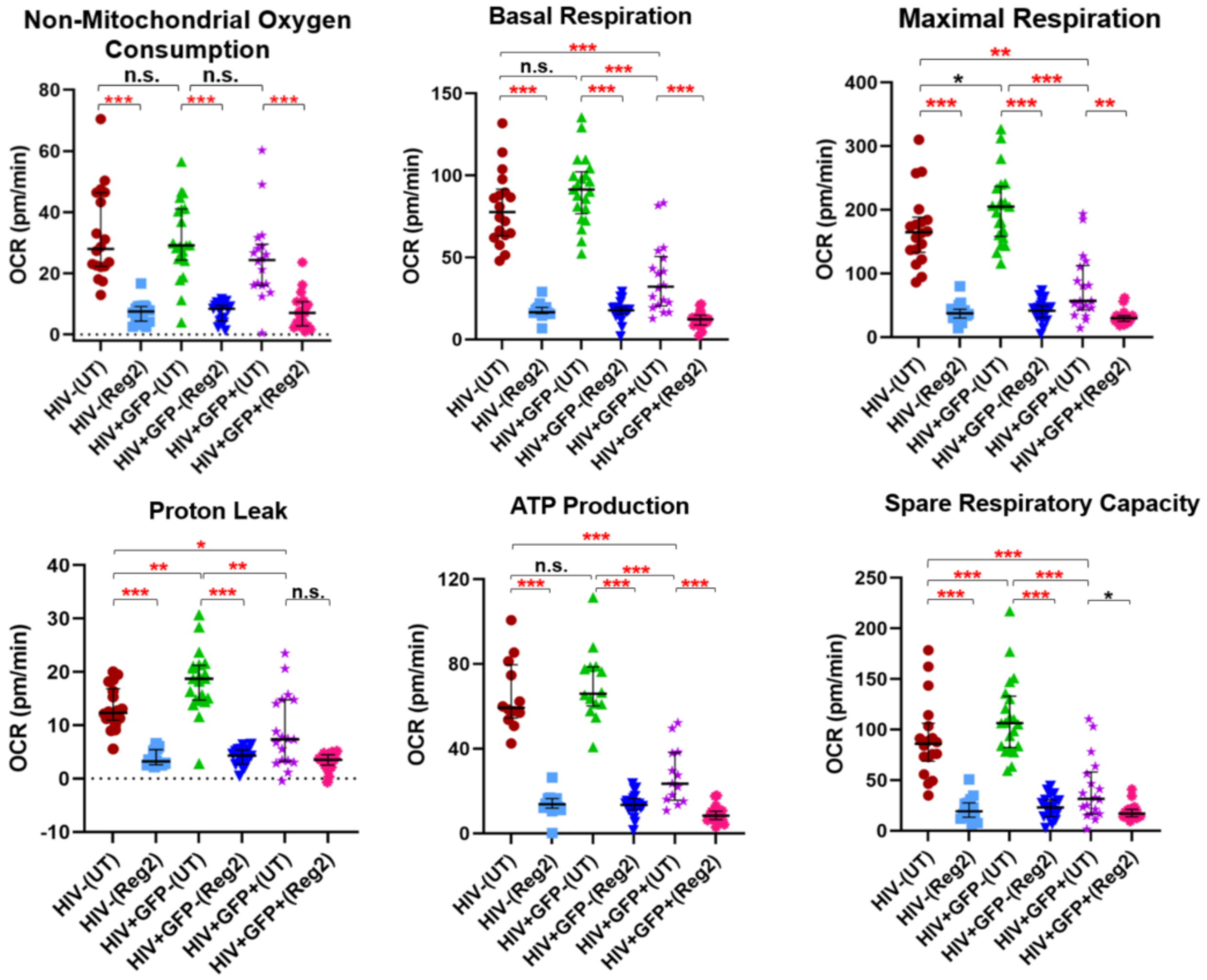

Figure S3. Differences in mitochondrial energy expenditure across uninfected, latently infected HIV+, and activated HIV+ hμglia groups before and after 24 hours of treatment with antiretroviral drugs. All components of mitochondrial respiration are shown. *Abbreviations: OCR*, oxygen consumption rate (pmol/min); *UT*, untreated (vehicle); *Reg2*, Regimen2 Statistical significance: *n.s*., *p*>0.10; **p*-value=0.05-0.09; **p*<0.05; ***p*<0.001; *******p<0.0001.

Basal OCR		
	N	Median (IQR)
HIV- UT	12	71.6 (66.7, 91.8)
HIV- Reg2	16	16.6 (15.9, 19.4)
HIV-GFP- UT	14	84.3 (77.5, 96.2)
HIV-GFP- Reg2	19	17.9 (15.6, 19.5)
HIV+GFP+ UT	13	31.2 (19.4, 43.3)
HIV+GFP+ Reg2	19	12.3 (8.8, 14.8)

Maximal OCR		
	N	Median (IQR)
HIV- UT	12	153.8 (133.8, 175.3)
HIV- Reg2	16	37.4 (30.3, 43.4)
HIV-GFP- UT	14	184.6 (159.5, 215.9)
HIV-GFP- Reg2	19	41.6 (30.8, 49.4)
HIV+GFP+ UT	13	52.5 (32.9, 80.2)
HIV+GFP+ Reg2	19	29.8 (24.7, 33.9)

Proton Leak		
	N	Median (IQR)
HIV- UT	12	11.9 (11.3, 14)
HIV- Reg2	16	3.2 (2.7, 5.3)
HIV-GFP- UT	14	16.6 (15.4, 19)
HIV-GFP- Reg2	19	4.3 (2.7, 5.4)
HIV+GFP+ UT	13	4.2 (2.9, 9.9)
HIV+GFP+ Reg2	19	3.5 (2.5, 4.5)

ATP Production		
	N	Median (IQR)
HIV- UT	12	59.2 (55.3, 77.9)
HIV- Reg2	16	13.8 (12, 16.3)
HIV-GFP- UT	14	66 (60.9, 78.6)
HIV-GFP- Reg2	19	13.5 (11.1, 16.5)
HIV+GFP+ UT	13	23.5 (16, 37.8)
HIV+GFP+ Reg2	19	8.3 (6.6, 10.5)

Spare Respiratory Capacity		
	N	Median (IQR)
HIV- UT	12	79.2 (67.0, 94.6)
HIV- Reg2	16	19.3 (13.6, 27.2)
HIV-GFP- UT	14	100.8 (81.7, 120.6)
HIV-GFP- Reg2	19	23.0 (14.1, 30.5)
HIV+GFP+ UT	13	25.0 (16.6, 36.9)
HIV+GFP+ Reg2	19	17.0 (13.9, 21.1)

Non-Mitochondrial OCR		
	N	Median (IQR)
HIV- UT	12	30.8 (22.5, 47.0)
HIV- Reg2	16	7.5 (4.6, 9.1)
HIV-GFP- UT	14	36.0 (25.2, 38.4)
HIV-GFP- Reg2	19	8.4 (4.4, 9.5)
HIV+GFP+ UT	13	26.7 (20.8, 32.4)
HIV+GFP+ Reg2	19	7.1 (2.8, 10.8)

Table S1. Comparison of mitochondrial respiratory measurements in uninfected, latently infected-HIV+, activated HIV+ hμglia with and without antiretroviral Regimen2 (*Reg2*) exposure for 24 hours. Medians and interquartile ranges (*IQR*) of oxygen consumption rate (OCR) values in pmol/min (normalized to total cellular protein) are shown. *p*-values for comparisons between vehicle-treated (*UT*) controls and treated cells of each type are provided in Figures 2-4 of the main text and in Figure S3. *HIV-,* uninfected; *GFP-*, latently infected-HIV+; *GFP+,* activated HIV+ cells.

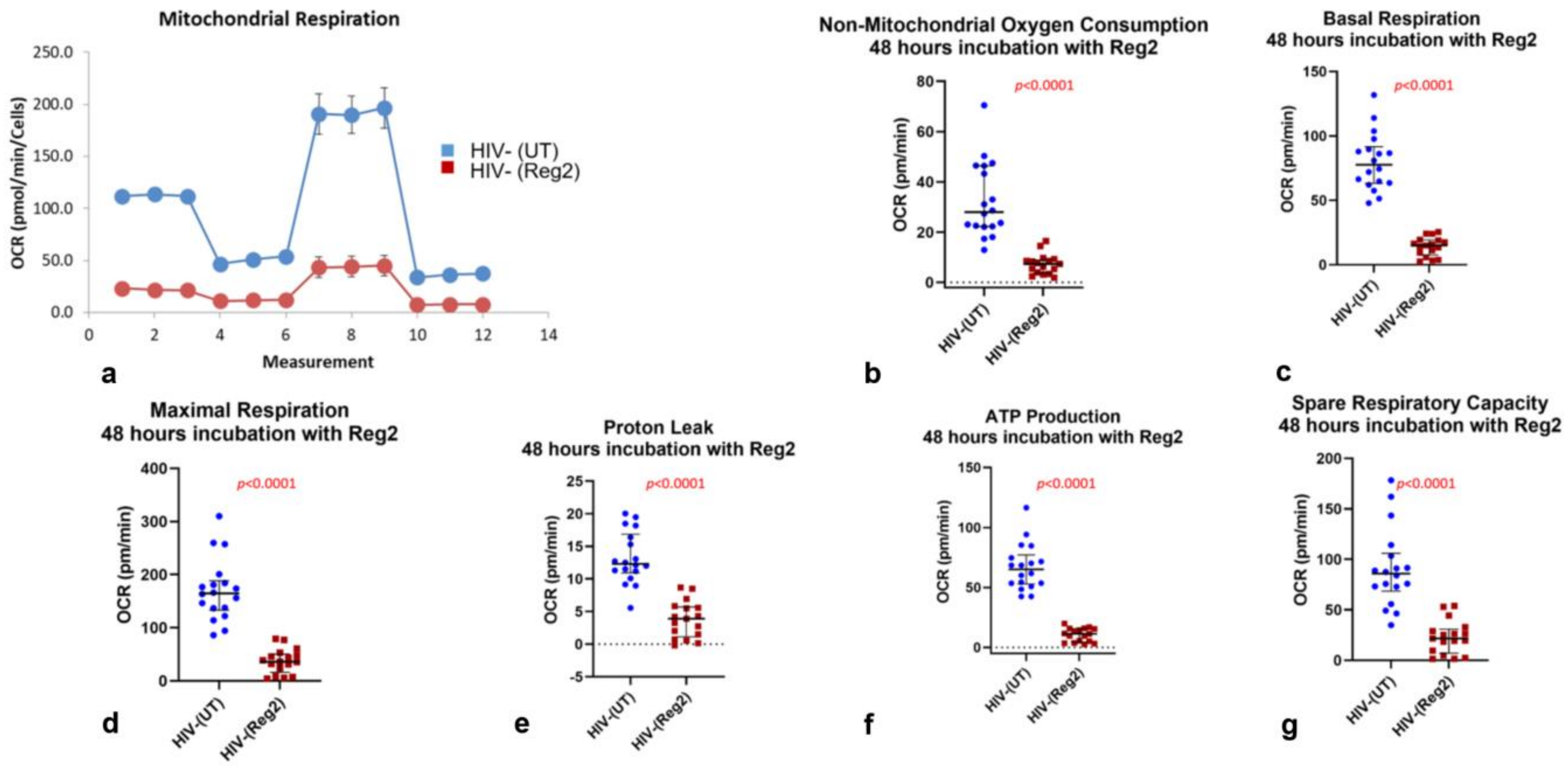

Figure S4. Mitochondrial bioenergetics measurements at 48 hours following Regimen2 exposure in uninfected hμglia. (**a**) MitoStress curve (blue=vehicle-treated controls (*UT*); red=drug-treated cells); (**b**) non-mitochondrial OCR; (**c**) basal respiration; (**d**) maximal respiration; (**e**) proton leak; (**f**) ATP production; (**g**) spare respiratory capacity. Median and interquartile ranges of OCR values obtained from at least six technical replicates and three separate experiments are shown. (*p*-values obtained by Mann-Whitney U test)

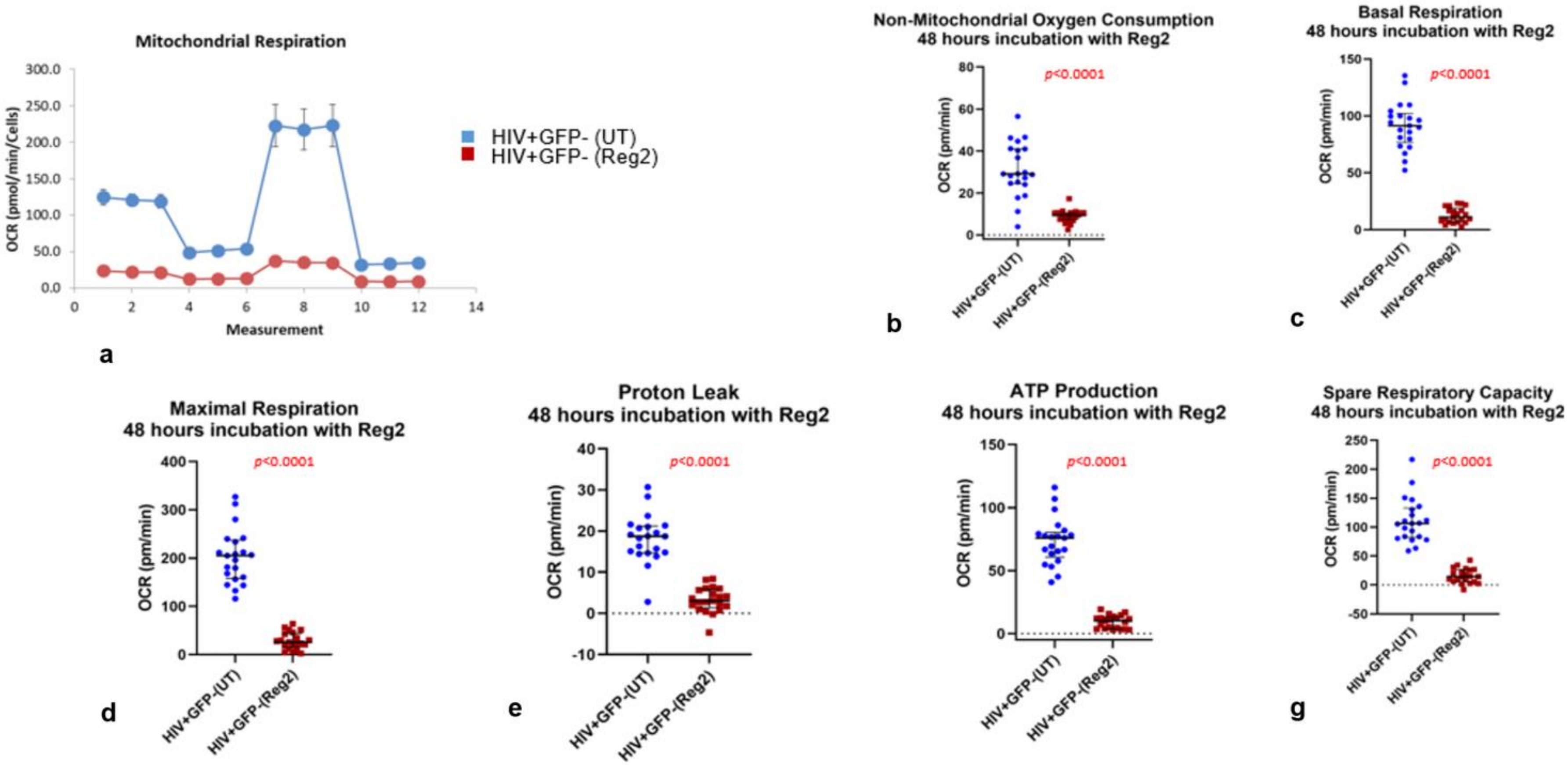

Figure S5. Antiretroviral Regimen2 (*Reg2*) reduces multiple components of mitochondrial respiratory function in latently infected-HIV+ hμglia at 48 hours. (**a**) MitoStress curve (blue=vehicle-treated controls (*UT*); red=drug-treated cells); (**b**) non-mitochondrial OCR; (**c**) basal respiration; (**d**) maximal respiration; (**e**) proton leak; (F) ATP production; (**g**) Spare respiratory capacity. Median and interquartile ranges of OCR values obtained from at least three separate experiments, each with six technical replicates, are shown. (*p*-values obtained by Mann-Whitney U test)

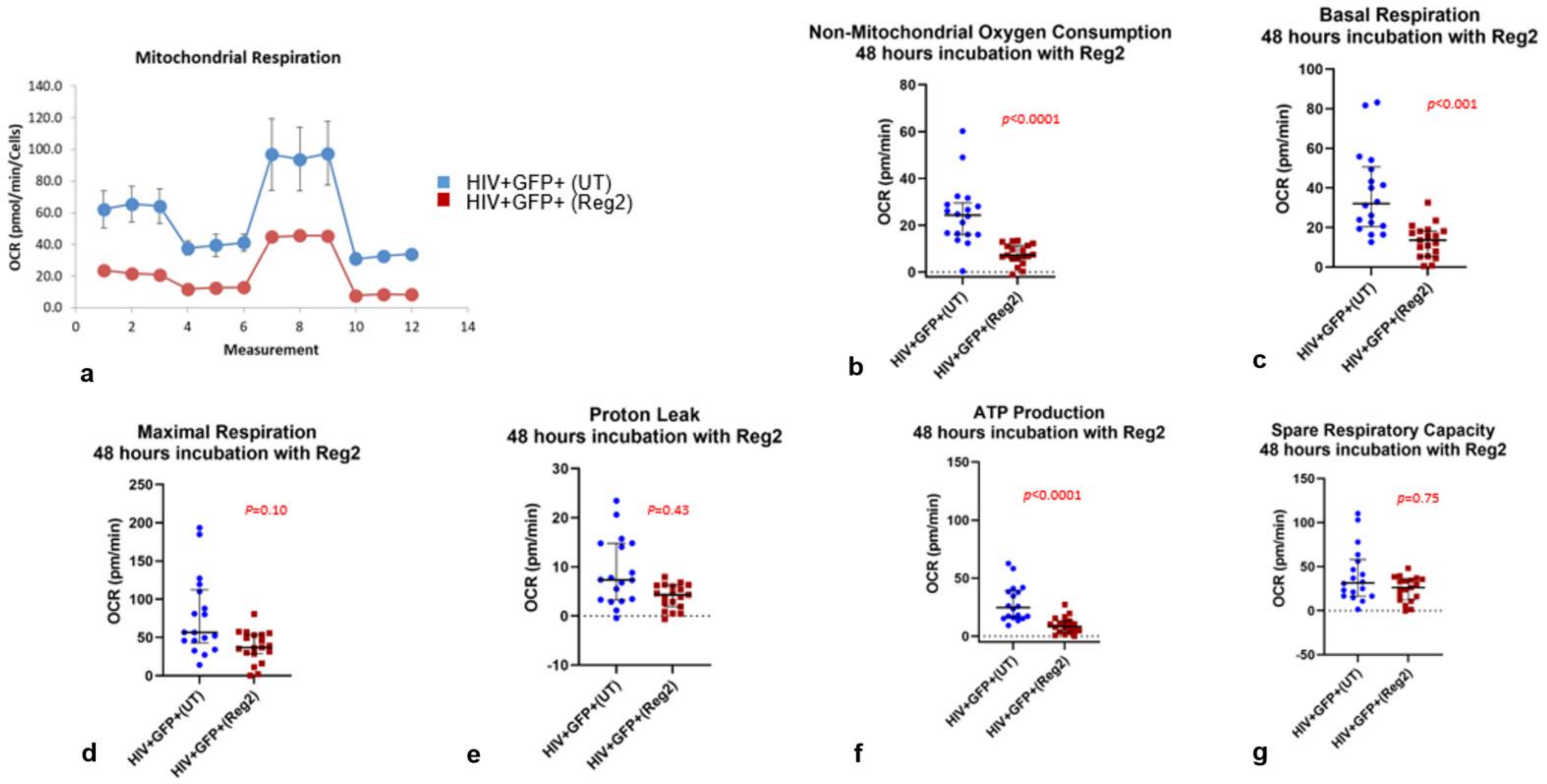

Figure S6. Antiretroviral Regimen2 (*Reg2*) reduces several components of mitochondrial respiratory function in activated HIV+ hµglia at 48 hours. (**a**), MitoStress curve (blue=vehicle-treated controls (*UT*); red=drug-treated cells); (**b**), non-mitochondrial OCR; (**c**) basal respiration; (**d**), maximal respiration; (**e**) proton leak; (**f**) ATP production; (**g**) spare respiratory capacity. Median and interquartile ranges of OCR values are shown. (*p*-values obtained by Mann-Whitney U test)

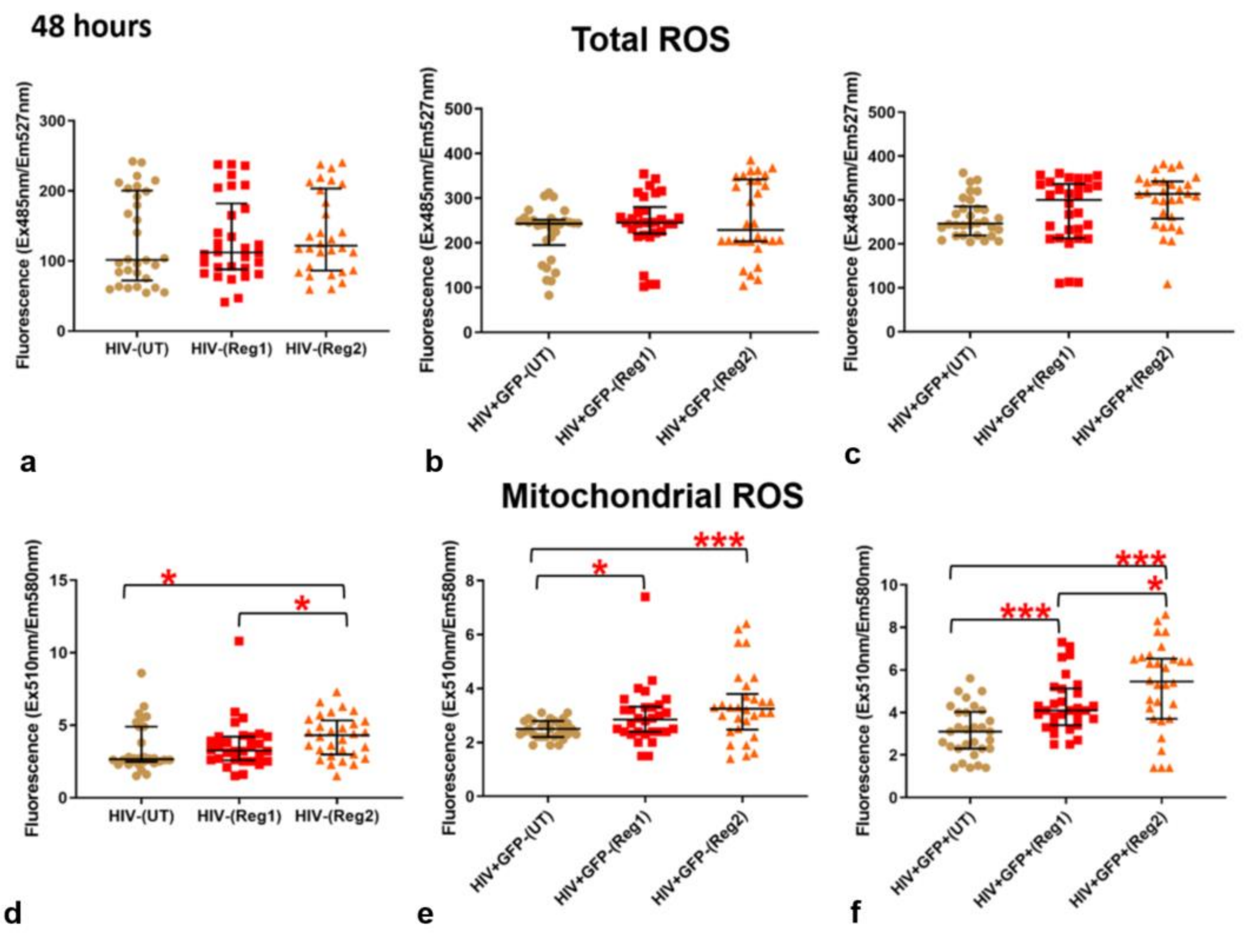

Figure S7. Effects of Regimen1 (*Reg1*) and Regimen2 (*Reg2*) after 48 hours on (**a-c**) total cellular reactive oxygen species (*ROS*) production and (**d-f**) mitochondrial ROS production in: (**a, d**) uninfected, (**b, e**) latently infected HIV+, and (**c, f**) activated HIV+ hμglia. **p*<0.05; ***p*<0.005;

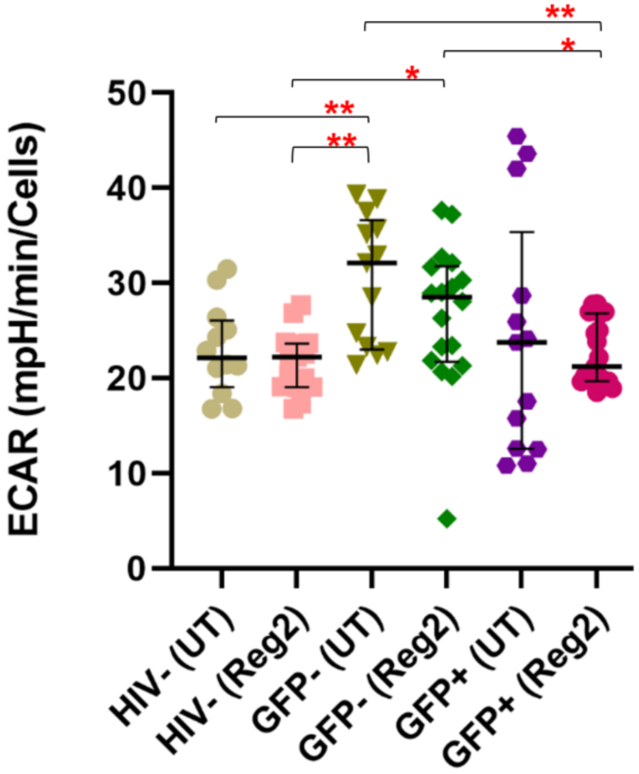

Figure S8. Extracellular acidification (glycolytic) rate (*ECAR*) measurements in uninfected, latently infected-HIV+(GFP-), and activated HIV+(GFP+) hμglia after a 24-hour incubation with Regimen2 (*Reg2*). Comparisons are made to untreated (vehicle) controls (*UT*), and only statistically significant differences are shown. *p<0.05; **p<0.005 (Mann-Whitney U test)

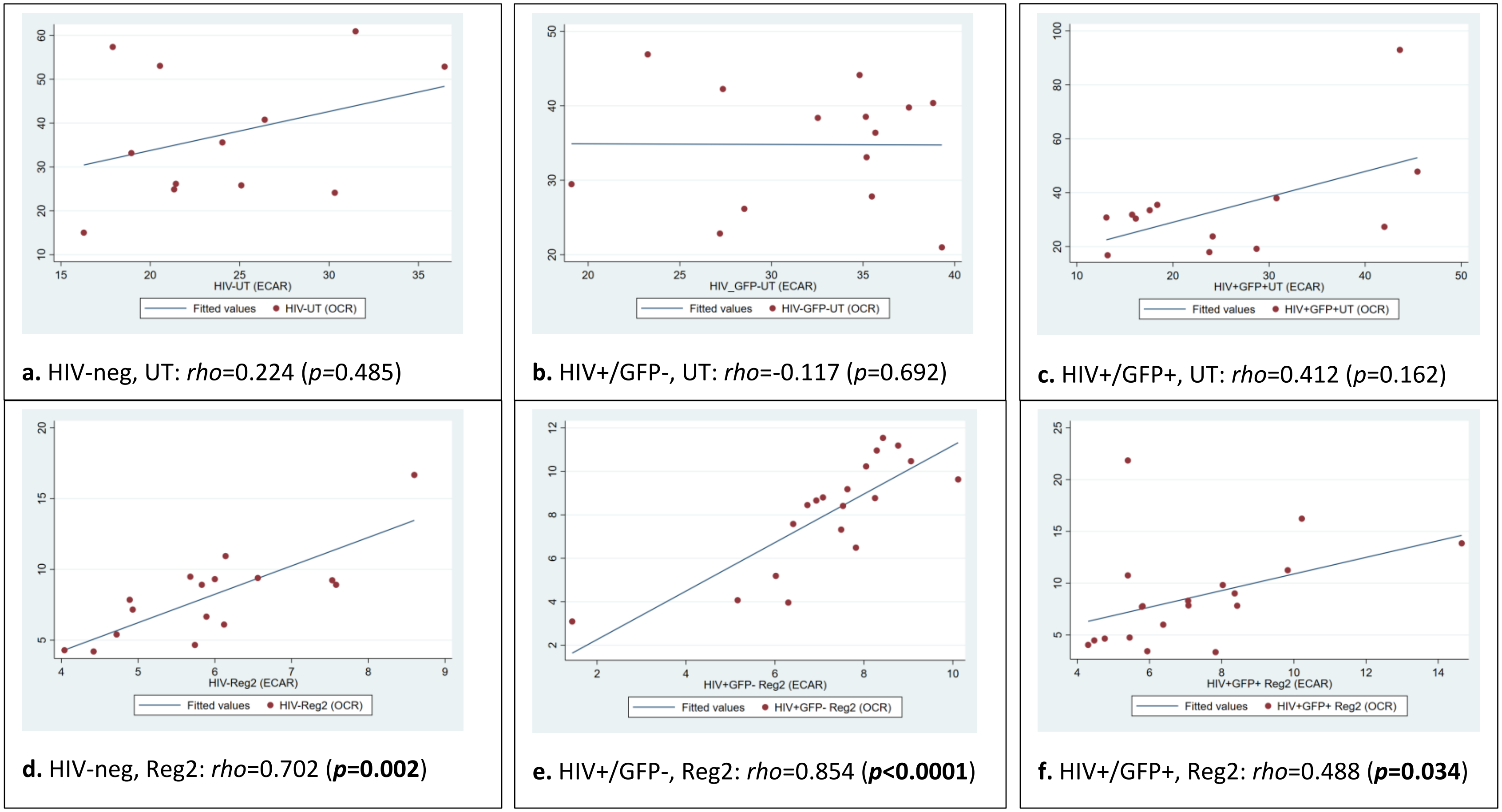

Figure S9. Correlations between basal OCR (plotted on the Y-axis) and ECAR measurements in uninfected *(HIV-neg)*, latently infected HIV+ *(HIV+/GFP-)*, and activated HIV+ *(HIV+/GFP+)* hμglia following Regimen2 exposure for 24 hours: (**a-c**) untreated cells; (**d-f**) Regimen2-treated cells after 24 hours. Spearman's *rho* correlation *p*-values<0.05 are statistically significant (**bolded**).

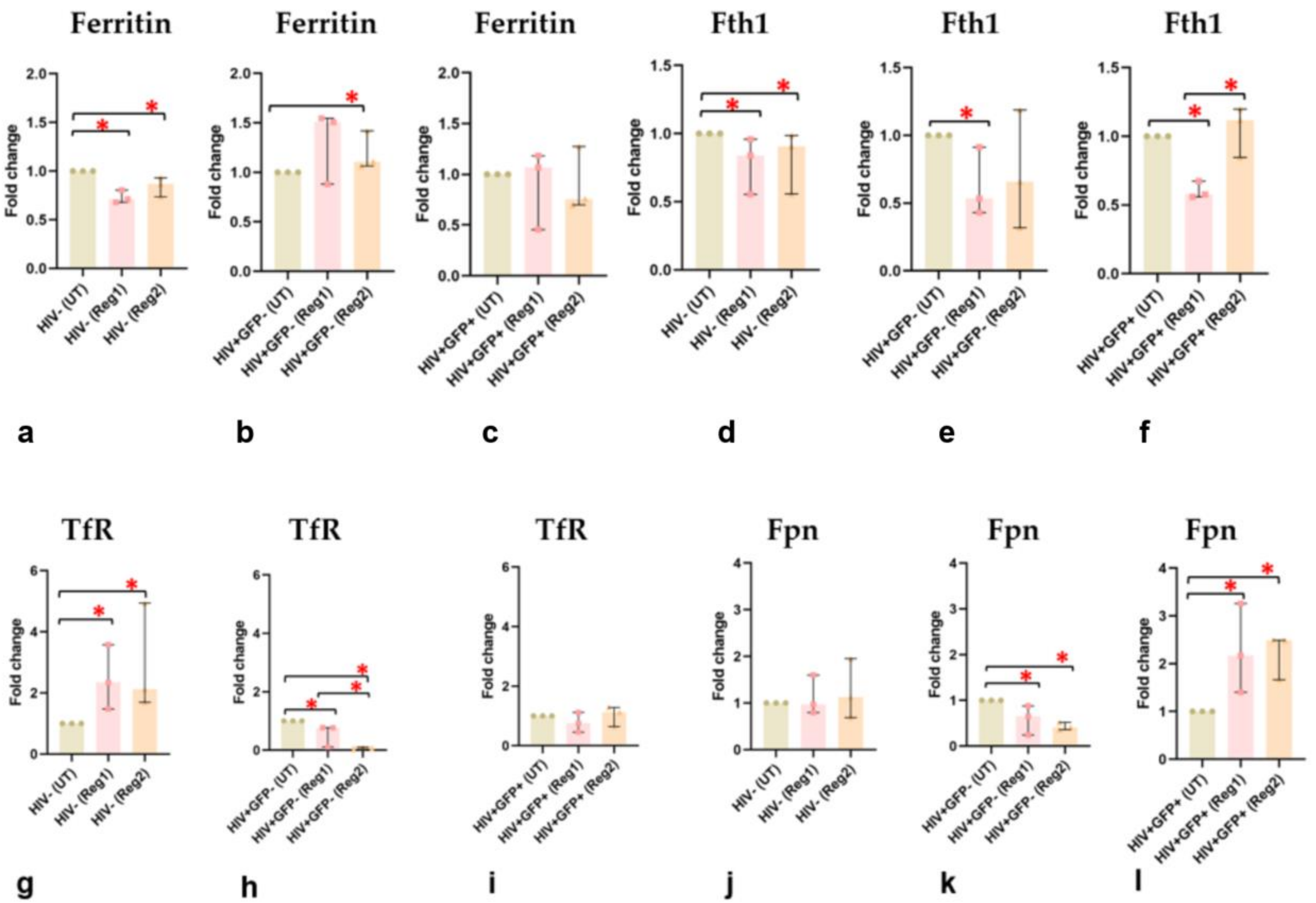

Figure S10. Quantification of (**a-c**) total ferritin, (**d-f**) Fth1, (**g-i**) TfR, and (**j-l**) Fpn in uninfected, latently infected HIV+ and activated HIV+ huglia at 48 hours after Regimen2 (*Reg2*) exposure. *UT*, untreated (vehicle-treated) controls; **p*<0.05 (Mann-Whitney U test)

Cell type	*Reg1*		*Reg2*		*Reg1 Iron phenotype*	*Reg2 Iron phenotype*
	24 hrs	*48 hrs*	*24 hrs*	*48 hrs*		
**HIV-negative**						
Ferritin	NC	DOWN*	NC	DOWN*	Iron import	Iron import
Fth1	NC	DOWN*	NC	DOWN*	Iron import	Iron import
TfR	UP*	UP*	UP*	UP*	Iron import	Iron import
Fpn	NC	NC	UP*	NC	None	Iron export
Iron	UP*	N/A	UP*	N/A	Iron replete/excess	Iron replete/excess
**Latent HIV+**						
Ferritin	UP*	NC	NC	DOWN*	Iron export	Iron import
Fth1	NC	DOWN*	NC	NC	Iron import	None
TfR	NC	DOWN*	NC	DOWN*	Iron export	Iron export
Fpn	NC	DOWN*	NC	DOWN*	Iron import	Iron import
Iron	UP*	N/A	UP*	N/A	Iron replete/excess	Iron replete/excess
**Activated HIV+**						
Ferritin	DOWN*	NC	DOWN*	NC	Iron import	Iron import
Fth1	NC	DOWN*	NC	UP*	Iron import	Iron export
TfR	NC	NC	NC	NC	None	None
Fpn	NC	UP*	NC	UP*	Iron export	Iron export
Iron	UP*	N/A	DOWN*	N/A	Iron replete/excess	Reduced/Low iron
**SH-SY5Y cells**	***BIC***	***Reg2***			**Comments**	
	24 hrs	*24 hrs*				
Ferritin	N/A	N/A			None	
Fth1	DOWN*	DOWN*			Iron import	
TfR	UP*	N/A			Iron import	
Iron	N/A	UP*			Iron replete/excess	

Table S2. Summary of changes in cellular iron phenotype of hµglia and SH-SY5Y cells after exposure to ARV Regimens 1 or 2, indicating altered iron homeostasis and dysregulated iron transport protein expression, due to both ARVs and HIV in hµglia, and ARVs in the neural cells. *Statistically significant with *p*-value<0.05; *Reg1/2,* Regimen 1/2; *NC*, no change; *N/A*, not assessed. TfR changes were used to anchor assessment of iron import *vs*. export.

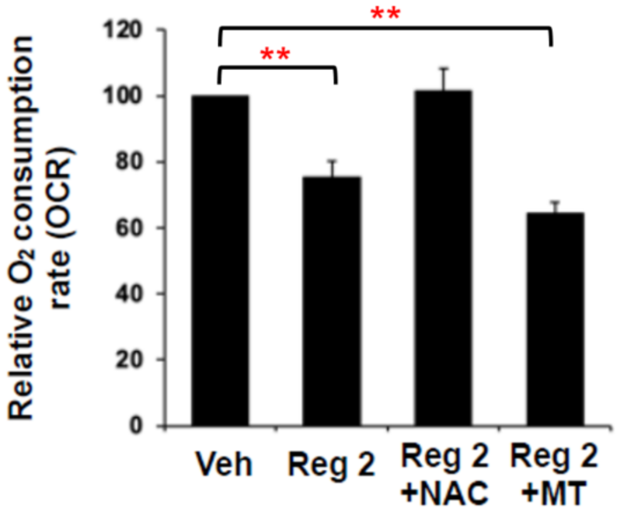

Figure S11. N-acetylcysteine (*NAC*), a cytosolic ROS scavenger, but not the mitochondrial ROS scavenger MitoTEMPO (*MT*) rescues the mitochondrial defect due to ARV Regimen2 (*Reg2*) in SH-SY5Y cells at 24 hours. **p=0.007
